# Chronic GIRK2 disruption in the weaver mouse selectively alters hippocampal excitability without affecting sharp wave-ripple dynamics

**DOI:** 10.3934/Neuroscience.2026024

**Published:** 2026-09-09

**Authors:** Giota Tsotsokou, Vasiliki Sarantopoulou, Costas Papatheodoropoulos

**Affiliations:** 1 Department of Medicine, Laboratory of Physiology, University of Patras, Greece; 2 School of Health Sciences, Laboratory of Experimental Animals, University of Patras, Greece

**Keywords:** hippocampus, dorsoventral axis, weaver mutation, sharp wave-ripple, network oscillations, neuronal synchronization, multiunit activity, excitation/inhibition balance, GIRK channels (Kir3.2), mice

## Abstract

Sharp wave-ripple complexes (SWRs) are intrinsic hippocampal network patterns fundamentally involved in memory processing and are sensitive to changes in excitation-inhibition balance. G protein-gated inwardly rectifying potassium (GIRK) channels regulate neuronal excitability, but their role in shaping SWR dynamics at the network level remains incompletely understood. The weaver mutation (Kcnj6^wv/wv^) disrupts the GIRK2 channel's function and provides a model of chronic GIRK alteration. Here, we examined how the weaver mutation affects SWR dynamics along the dorsoventral hippocampal axis and whether these effects resemble those of acute GIRK blockade. Extracellular local field potentials and multiunit activity (MUA) were recorded from dorsal and ventral hippocampal slices of wild-type (WT) and weaver mice. SWR incidence, amplitude, ripple properties, and SWR-associated firing were quantified. The effects of the GIRK blocker tertiapin-Q were also assessed. SWR occurrence was strongly determined by dorsoventral location, with higher event rates in the ventral hippocampus, and was not affected by genotype. Similarly, SWR amplitude was region-dependent and preserved in weaver mice. Ripple oscillation properties were largely preserved, with no consistent genotype-dependent differences. In contrast, baseline neuronal activity (MUA-Base) was significantly increased in weaver mice, indicating enhanced intrinsic excitability, whereas SWR-associated firing (MUA-SWR) showed a tendency toward reduction. Acute GIRK blockade with tertiapin-Q did not significantly alter SWR parameters in either genotype, nor did it interact with genotype effects. Together, these findings indicate that chronic GIRK disruption produces selective alterations in the hippocampal network's function that are not mimicked by acute pharmacological inhibition of these channels. While the basic features of SWR generation are preserved, increased baseline excitability and altered neuronal recruitment suggest circuit-level adaptations that reshape hippocampal dynamics beyond direct GIRK channel effects.

## 1. Introduction

Sharp wave-ripple complexes (SWRs) are highly synchronous hippocampal population events that emerge from the interaction of recurrent excitation in Cornu Ammonis 3 (CA3) and perisomatic inhibition in Cornu Ammonis 1 (CA1), giving rise to a large-amplitude sharp wave with superimposed fast ripple oscillations (100–200 Hz) in the local field potential [1]. They occur predominantly during quiet wakefulness and non-rapid eye movement (REM) sleep and are critically involved in memory consolidation, planning, and hippocampal-cortical communication, supporting the reactivation and reorganization of behaviorally relevant neuronal assemblies [1]–[4]. Because SWRs depend on tightly coordinated interactions between excitatory pyramidal neurons and inhibitory interneuron networks, they are highly sensitive to changes in excitation-inhibition balance and provide a valuable assay for circuit-level dysfunction [1],[5]–[9].

A major organizational feature of the hippocampus is its functional specialization along the dorsoventral (septotemporal) axis. The dorsal hippocampus primarily supports spatial and cognitive functions, whereas the ventral hippocampus is more strongly involved in affective regulation and stress-related processing through preferential connectivity with limbic and hypothalamic structures [10]–[13]. These functional distinctions are paralleled by systematic differences in intrinsic excitability, connectivity, and neuromodulatory tone, which shape region-specific oscillatory dynamics [14]. Consistent with this organization, SWRs exhibit robust dorsoventral heterogeneity both in vivo and in vitro [7],[15]–[20].

SWRs are particularly sensitive to perturbations of intrinsic excitability and neuronal synchronization, making them a valuable tool for probing the functional consequences of genetic mutations that alter the network's organization. In this context, the weaver (WV) mice (Kcnj6^wv/wv^) represent a well-characterized model of altered neuronal excitability and circuit dysfunction. The WV mutation provides a well-characterized model of disrupted function of G protein-gated inwardly rectifying potassium (GIRK) channels, a subfamily of Kir channels [21],[22], which are key regulators of neuronal excitability and synaptic integration, acting as major effectors of inhibitory neuromodulatory signaling, thereby influencing neuronal excitability, synaptic plasticity and contributing to the stability of network activity and cognitive function [22]–[26]. Specifically, the WV phenotype results from a G156S mis-sense point mutation in the pore-forming region of the GIRK2 (Kir3.2) channel, which alters ion selectivity and disrupts normal membrane properties, leading to increased neuronal excitability and altered network behavior [24],[27]. Given their role in shaping neuronal excitability, GIRK channels are well positioned to influence emergent network events such as SWRs. In the hippocampus, GIRK2-containing channels are expressed by both pyramidal neurons and several classes of inhibitory interneurons, where they regulate membrane excitability and synaptic integration [22],[24],[28]. Notably, GIRK channels' expression and function also exhibit regional variation along the dorsoventral axis [29],[30], suggesting a potential contribution to regional differences in network dynamics.

Beyond altered GIRK function, the WV mutation produces widespread neurodevelopmental abnormalities, including disrupted neuronal migration, long-term synaptic alterations, and changes in network organization [31]–[33]. Genetic perturbations of ion channels often trigger compensatory and homeostatic mechanisms during development, leading to circuit-level reorganization [34]–[36]. Because SWRs emerge from coordinated population activity, they are particularly sensitive to both direct conductance changes and secondary network adaptations.

Despite extensive characterization of the WV phenotype, its impact on hippocampal population dynamics and SWR organization remains poorly understood. Given that GIRK channels modulate neuronal excitability and that SWRs critically depend on excitation/inhibition balance and neuronal synchronization, disruption of GIRK signaling is expected to influence the hippocampal network's dynamics, although the extent and specificity of these effects at the population level remain unclear. To address this issue, we examined spontaneous SWRs and associated multiunit activity in dorsal and ventral hippocampal slices from wild-type (WT) and WV mice. By combining a genetic model with a dorsoventral comparative approach and pharmacological manipulation, we aimed to determine whether chronic disruption of GIRK2 signaling produces region-specific alterations in SWR dynamics and whether these effects can be recapitulated by acute GIRK channel blockade.

## 2. Materials and methods

### 2.1. Animals

Experiments were conducted using 1.5–2-month-old male and female homozygous WV mice (A-Kcnj6^wv/wv^) and corresponding WT controls of the B6CBACa background. Animals were housed in a pathogen-free facility under controlled environmental conditions (12 h light/dark cycle; 20–22 °C) with ad libitum access to food and water. All procedures complied with European guidelines for animal care (Directive 86/609/EEC) and were approved by the institutional and regional regulatory authorities (University of Patras and Veterinary Services of Western Greece; Reg. No. 109724/403/2020). Ethical oversight was additionally provided by the University Animal Welfare Committee. Homozygous WV mice were identified postnatally (P14) on the basis of the characteristic motor phenotype (ataxia, tremor, gait instability) [37]. Breeding was performed using heterozygous pairs, as homozygous males are infertile. Genotyping of breeders was confirmed by polymerase chain reaction (PCR) using standard amplification protocols: (1) Pro-incubation (95 °C, 15 min), (2) denaturation (95 °C, 30 s), (3) annealing (54.5 °C, 30 s), (4) extension (72 °C, 1 min), and (5) final step (72 °C, 10 min). Sample size was determined using power analysis (G*Power).

### 2.2. Slice preparation and maintenance

Following deep anesthesia, brains were rapidly removed and transferred into ice-cold, oxygenated artificial cerebrospinal fluid (ACSF). The ACSF's composition was 124 mM NaCl, 4 mM KCl, 2 mM CaCl_2_, 2 mM MgSO_4_, 26 mM NaHCO_3_, 1.25 mM NaH_2_PO_4_, and 10 mM glucose. Transverse hippocampal slices (500 µm) were obtained from dorsal and ventral regions using a tissue chopper. Slices were maintained in an interface recording chamber under continuous perfusion with oxygenated ACSF (95% O_2_/5% CO_2_; ~1.5 mL/min) at 30 ± 0.5 °C. Recordings commenced after at least 90 min of recovery. The high-affinity blocker for inward rectifier potassium channels tertiapin-Q (Tocris Cookson Ltd., Bristol, UK; 50 nM) was used in this study to block GIRK channels (Kir1.1, Kir3.1, and Kir3.4 inwardly rectifying potassium channels) [38]. Slices were perfused with tertiapin-Q (TPN-Q) for 30 min.

### 2.3. Electrophysiology and signal processing

Extracellular field potentials were recorded from the CA1 stratum pyramidale using carbon fiber electrodes. Signals were amplified, filtered (0.5 Hz–2 kHz), and digitized at 10 kHz. Spontaneous activity consisted of SWR events and associated multiunit activity (MUA). Signals were downsampled (1 kHz) and low-pass-filtered (<35 Hz) to isolate the sharp wave component. SWRs were detected using threshold-based algorithms and confirmed by visual inspection. The following parameters were quantified: Interevent interval (IEI), SWR amplitude, ripple peak frequency, and ripple power. Spectral analysis was performed using fast Fourier transform (FFT) with a Hanning window (1.638 s; ~0.61 Hz resolution). Ripple features were extracted within the 75–250 Hz band. MUA was extracted from high-frequency signals (400–1500 Hz) using threshold detection. Neuronal firing associated with SWRs was estimated by preparing peri-event time histograms of MUA by aligning detected spikes to the peak of each sharp wave event. The SWR-associated MUA (MUA-SWR) was defined as the peak firing rate within the peri-event histogram centered on the sharp wave. All measures were obtained from stable recording periods (last 5 min per condition).

### 2.4. Statistical analysis

The experimental unit in this study was the hippocampal slice. Data were analyzed using linear mixed-effects models (LMMs) to account for the hierarchical structure of the dataset. For genotype-related analyses, the fixed effects included genotype (WT vs. WV), the hippocampal region (dorsal vs ventral), and their interaction (genotype × region). A random intercept was included to account for variability across slices. Given the limited number of slices per animal and the variability across recordings, animal identity was not included as a random factor. For pharmacological experiments, analyses were restricted to control and tertiapin-Q conditions, excluding washout to avoid unequal group sizes and potential carry-over effects. The LMMs included the drug condition (control vs tertiapin-Q), genotype, and their interaction as fixed effects, with slice included as a random factor to account for repeated measurements within slices. Model assumptions (normality and homogeneity of residuals) were assessed by visual inspection of the residual plots. When necessary, logarithmic transformation was applied to improve normality and stabilize variance (e.g., inter-event intervals and ripple power). Estimated marginal means were computed for descriptive purposes. Because no significant interaction effects were detected in the final models, no post hoc pairwise comparisons were performed. For visualization, data are presented as diamond plots, showing the median, interquartile range (25^th^–75^th^ percentiles), mean, and 5^th^–95^th^ percentiles; individual data points represent slices. Statistical significance was set at p < 0.05. Additional analyses were performed to characterize the variability and temporal organization of spontaneous SWRs. The variability of IEIs and SWR amplitude was quantified for each hippocampal slice as the coefficient of variation (CV = SD/mean × 100) and analyzed using LMMs. The relationship between the IEI preceding each SWR and the amplitude of the subsequent SWR was assessed by calculating Pearson's correlation coefficient separately for each slice. Correlation coefficients were transformed using Fisher's *z* transformation before statistical comparison between groups using LMMs.

## 3. Results

We analyzed spontaneous SWR activity and the associated multiunit firing in hippocampal slices obtained from WT and WV mice. Recordings were performed in CA1 and included both dorsal and ventral hippocampal regions (Figure 1A). From these recordings, we quantified the principal features of spontaneous SWRs, including IEI and amplitude (Figure 1B,C), ripple power and peak frequency (Figure 1D–F), and baseline MUA (MUA-Base) and MUA-SWR (Figure 1G–I).

### 3.1. SWR occurrence is determined by the dorsoventral location, not genotype

Consistent with previous work demonstrating the strong dorsoventral organization of SWRs, analyses were conducted both at the regional level and after pooling across the longitudinal axis to assess genotype-dependent effects. Analysis of the IEIs revealed a strong effect of dorsoventral location (F_(1,75)_ = 28.01, p < 0.001; Figure 1B), with shorter IEIs (higher SWR rates) observed in the ventral hippocampus compared with dorsal regions. In contrast, no significant effect of genotype was detected (F_(1,75)_ = 0.61, p = 0.439; Figure 2A), and the interaction between genotype and region was not significant (F_(1,75)_ = 1.60, p = 0.210), indicating that the dorsoventral organization of SWR occurrence is preserved in WV mice. The results of the LMM analyses are summarized in Table 1. Inspection of the raw data (Figure 1B) suggested greater variability in IEI values in dorsal WV hippocampus. This observation was examined quantitatively by analyzing the coefficient of variation (Section 3.3).

### 3.2. SWR amplitude is determined by dorsoventral location

SWR amplitude was significantly influenced by hippocampal region (F_(1,75)_ = 5.07, *p* = 0.027), with higher amplitudes observed in the ventral compared with the dorsal hippocampus (Figure 1C). In contrast, no main effect of genotype was detected (F_(1,75)_ = 0.91, *p* = 0.344; Figure 2B), and the interaction between genotype and region was not significant (F_(1,75)_ = 2.30, *p* = 0.134), indicating that amplitude differences were primarily region-dependent and preserved across genotypes (Table 1).

### 3.3. Variability of SWR occurrence and amplitude

To further characterize spontaneous SWR dynamics, we quantified the coefficient of variation (CV) of IEIs and SWR amplitude for each hippocampal slice. LMM analysis revealed a significant main effect of hippocampal region on the CV of IEIs (F_(1,75)_ = 12.78, p < 0.001), whereas neither genotype nor the interaction between genotype and region reached statistical significance (Supplementary Figure S1A). Analysis of the coefficient of variation of SWR amplitude revealed no significant effects of genotype, hippocampal region, or their interaction (Supplementary Figure S1B).

**Table 1.** Effects of genotype and dorsal/ventral region on SWR and MUA parameters.

Variable	Effect	F_(df1, df2)_	p
IEI	Genotype	F_(1,75)_ = 0.61	0.439
	Region	F_(1,75)_ = 28.01	**<0.001**
	Interaction	F_(1,75)_ = 1.60	0.210
Amplitude	Genotype	F_(1,75)_ = 0.91	0.344
	Region	F_(1,75)_ = 5.07	**0.027**
	Interaction	F_(1,75)_ = 2.30	0.134
Ripple frequency	Genotype	F_(1,72)_ = 2.70	0.105
Ripple power	Genotype	F_(1,26)_ = 2.87	0.102
MUA-base	Genotype	F_(1,69)_ = 4.99	**0.029**
	Region	F_(1,69)_ = 2.32	0.132
	Interaction	F_(1,69)_ = 0.34	0.560
MUA-SWR	Genotype	F_(1,70)_ = 2.90	0.093
	Region	F_(1,70)_ = 37.30	**<0.001**
	Interaction	F_(1,70)_ = 0.48	0.489

LMMs were run with genotype (WT vs. WV) and region (dorsal vs. ventral) as factors. Values represent F statistics with the corresponding p-values. Significant effects are indicated in bold.

**Figure 1.**
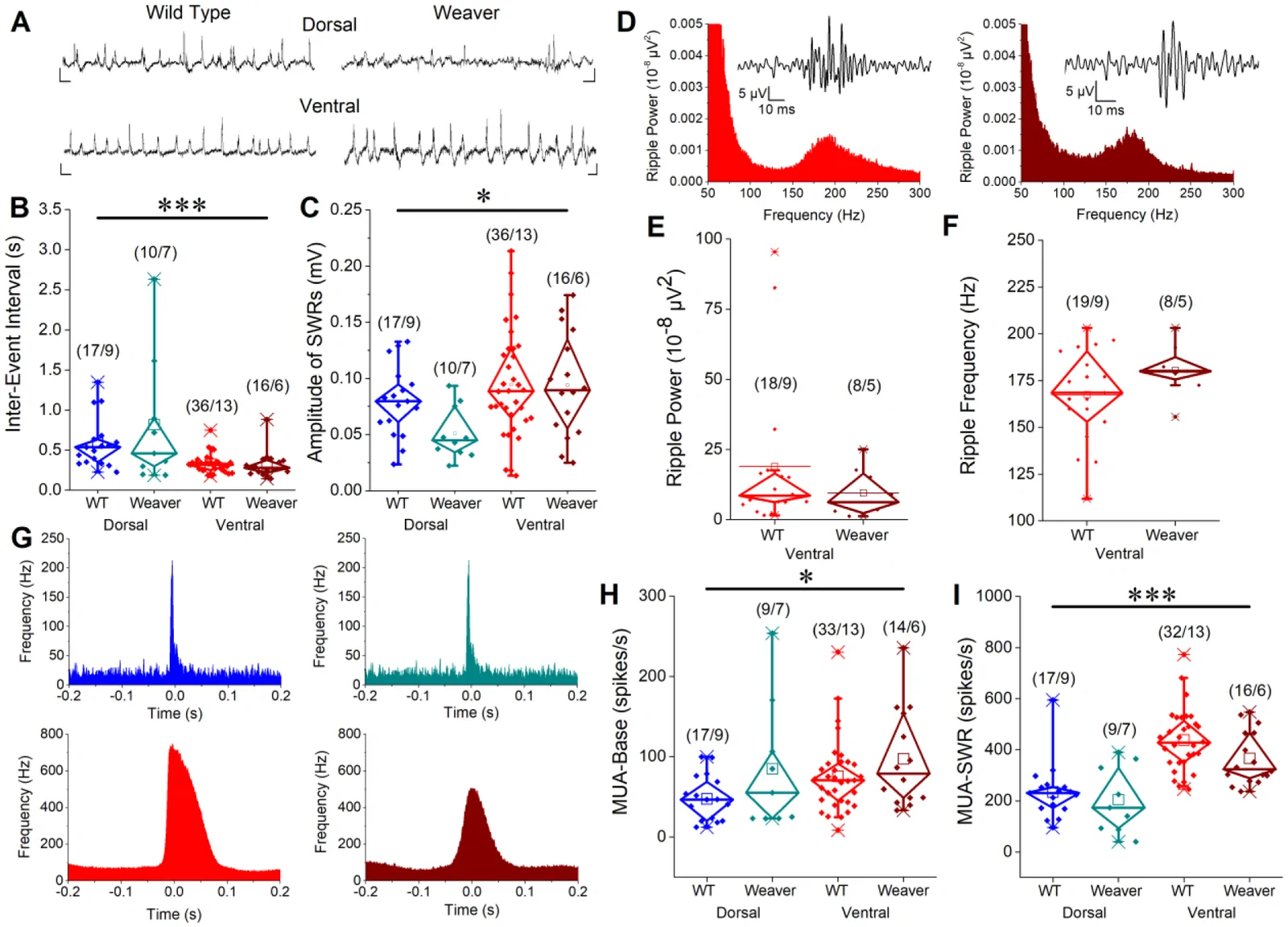
Dorsoventral organization of SWR properties is preserved in WV mice. A: Representative local field potential recordings from dorsal (DH) and ventral (VH) hippocampal slices of WT and WV mice showing spontaneous SWRs. Scale bars: 0.1 mV, 100 ms. B: IEI. IEI differed significantly between hippocampal regions (dorsal vs ventral), with shorter IEIs in ventral CA1, but did not differ between genotypes and showed no interaction between genotype and region. C: SWR amplitude. Amplitude was significantly higher in ventral compared with dorsal hippocampus and was not affected by genotype; no genotype × region interaction was observed. D: Representative ripple power spectra and corresponding ripple waveforms. E: Ripple power (ventral hippocampus). No significant difference between WT and WV slices (log-transformed data), although a tendency toward lower values in WV was observed. F: Ripple peak frequency (ventral hippocampus). No significant difference between genotypes. G: Time–frequency representations of SWR-associated activity (MUA-SWR) in dorsal and ventral hippocampus for WT and WV slices. H: Baseline multiunit activity (MUA-Base). WV slices exhibited significantly higher baseline firing rates compared with WT, independent of region. I: SWR-associated multiunit activity (MUA-SWR). No significant effect of genotype was detected, although a tendency toward reduced firing during SWRs was observed in WV slices; a strong dorsoventral difference was present, with higher values in ventral hippocampus. Data are presented as diamond plots showing median, interquartile range, mean, and 5^th^–95^th^ percentiles; individual data points represent slices. Numbers in parentheses indicate the number of slices/animals per group. Asterisks indicate significant main effects identified by LMMs (*p < 0.05; ***p < 0.001): Region effects in B, C, and I, and genotype effect in H.

### 3.4. Ripple oscillation properties are not significantly altered by genotype

Ripple properties were analyzed in the ventral hippocampus (Figure 1D–F). Ripple power did not differ significantly between genotypes (F_(1,26)_ = 2.87, p = 0.102; Figure 2C), although a tendency toward reduced power was observed in WV mice. Similarly, ripple peak frequency was not significantly affected by genotype (F_(1,72)_ = 2.70, p = 0.105; Figure 2D and Table 1). These findings indicate that the core oscillatory features of SWRs are largely preserved.

### 3.5. Dissociation between baseline excitability and SWR-related neuronal recruitment

MUA-Base was significantly increased in WV mice compared with WT mice (F_(1,69)_ = 4.99, p = 0.029; Figure 2E), indicating enhanced intrinsic neuronal excitability (Fig. 1G,H). No significant effect of dorsoventral region or the interaction between factors was observed. In contrast, SWR-associated neuronal firing (MUA-SWR) did not differ significantly between genotypes (F_(1,70)_ = 2.90, p = 0.093; Figure 2F), although a consistent reduction was observed in WV mice across both regions (Figure 1G,I). A strong effect of dorsoventral location was detected (F_(1,70)_ = 37.30, p < 0.001), with higher firing rates in the ventral hippocampus (Figure 1I and Table 1).

**Figure 2.**
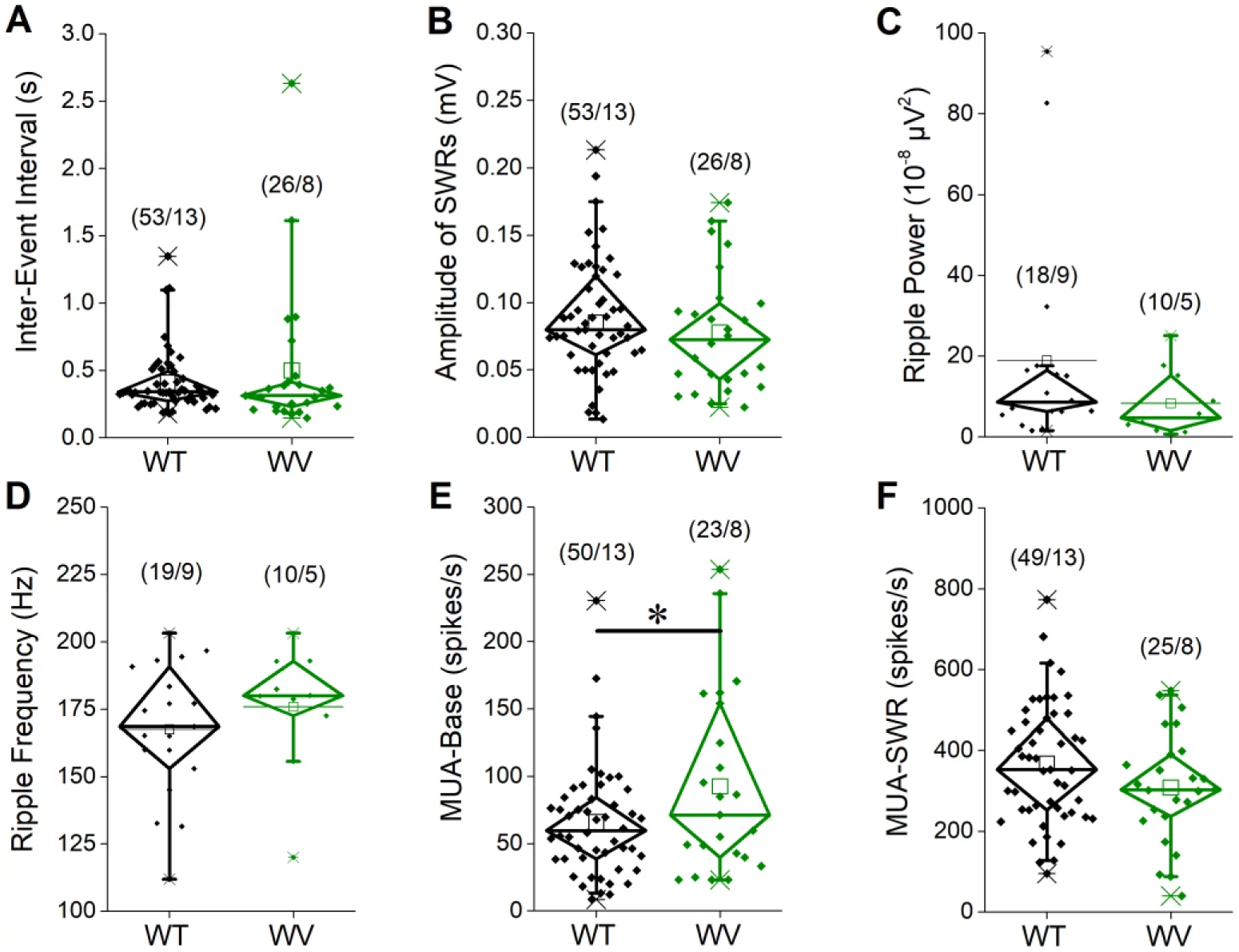
Genotype-dependent comparison of SWR and MUA properties at the whole hippocampus level. A: IEI of SWRs. No significant difference was seen between WT and WV mice. B: SWR amplitude. A trend toward reduced amplitude is observed in WV mice compared with WT. C: Ripple power. No significant difference was seen between WT and WV slices (log-transformed data), although a tendency toward lower values in WV is observed. D: Ripple peak frequency. No significant difference was seen between genotypes. E: Baseline multiunit activity (MUA-Base). WV slices exhibit significantly higher baseline firing rates compared with WT slices. F: MUA-SWR. No significant difference was seen between genotypes, although a tendency toward reduced firing during SWRs is observed in WV slices. Data are presented as diamond plots showing the median, interquartile range, mean, and 5^th^–95^th^ percentiles; individual data points represent slices, and numbers in parentheses indicate the number of slices/animals per group. An asterisk indicates a significant difference at p < 0.05.

### 3.6. Relationship between SWR timing and amplitude

To determine whether the temporal spacing of spontaneous SWRs influences the magnitude of the subsequent event, we analyzed the relationship between the preceding IEI and SWR amplitude. Pooling all detected SWRs revealed a weak but significant positive correlation between IEI and SWR amplitude (Pearson's r = 0.178, p < 0.0001; Figure 3A), indicating that longer intervals tended to precede slightly larger SWRs. To assess whether this relationship differed between genotypes, Pearson correlation coefficients were calculated for each slice, transformed using Fisher's z, and compared using LMM analysis. No significant genotype effect was detected (Figure 3B), indicating that the coupling between SWR timing and amplitude was preserved in WV mice.

**Figure 3.**
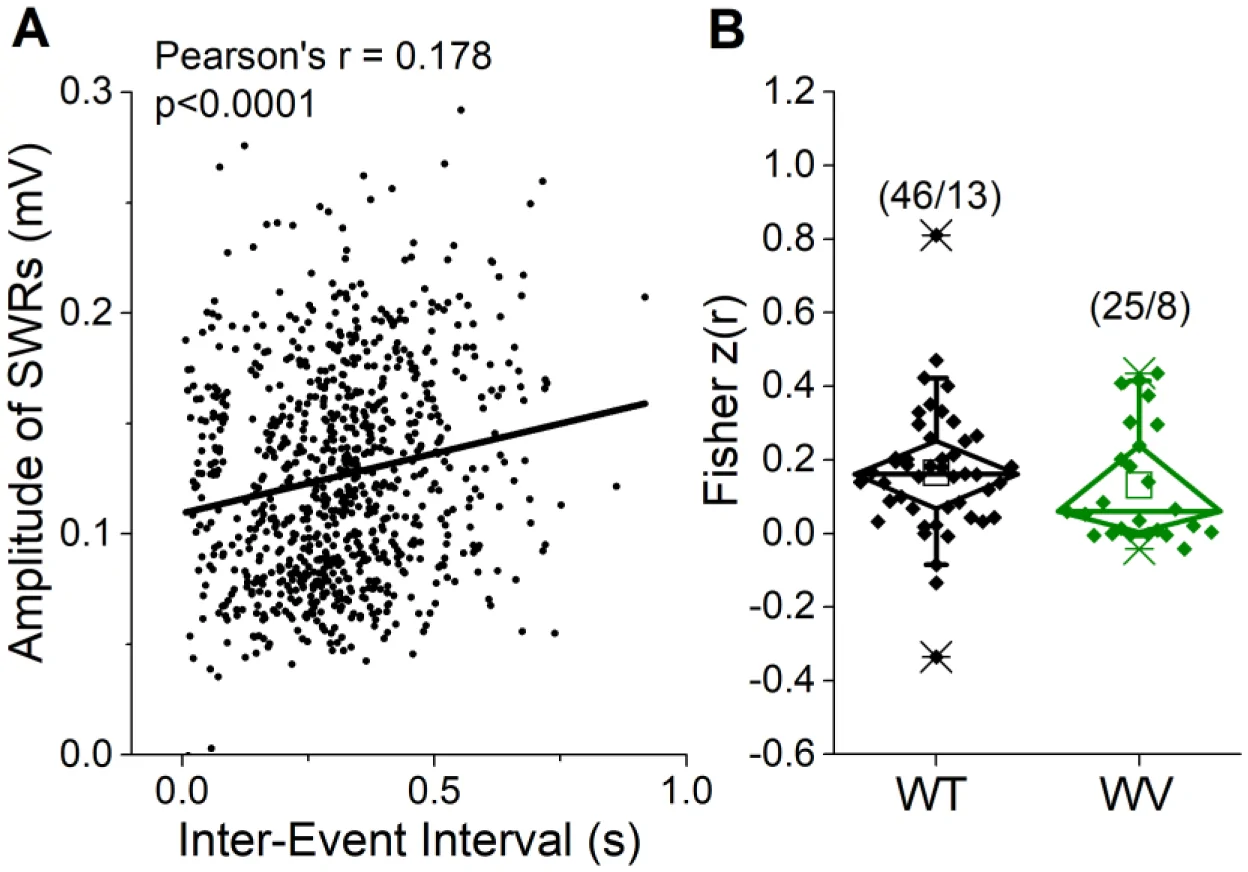
Relationship between SWR timing and amplitude. A. Scatter plot showing the relationship between the IEI preceding each SWR and the amplitude of the subsequent SWR for all detected SWRs. The regression line indicates a weak but significant positive correlation (Pearson's r = 0.178, p < 0.0001). B. Slice-wise Pearson correlation coefficients were transformed using Fisher's z and compared between WT and WV mice. No significant genotype effect was observed. Diamond plots show the median, interquartile range, mean, 5^th^–95^th^ percentiles, and outliers. Numbers indicate slices/animals.

Together, these results reveal a selective reorganization of the hippocampal network's dynamics in the WV model. While the dorsoventral organization of SWR occurrence is preserved, baseline neuronal activity is increased globally, and SWR-associated recruitment shows a tendency toward reduction. In contrast, SWR amplitude and ripples' oscillatory features remain largely preserved. This pattern indicates a dissociation between increased baseline excitability and impaired coordination of neuronal ensembles during network events.

**Table 2.** Effects of tertiapin-Q on SWR and MUA parameters.

Variable	Effect	F_(df1, df2)_	p
IEI	Genotype	F_(1,52)_ = 0.14	0.710
	Condition	F_(1,52)_ = 1.12	0.295
	Interaction	F_(1,52)_ = 0.17	0.681
Amplitude	Genotype	F_(1,52)_ = 0.07	0.788
	Condition	F_(1,52)_ = 0.40	0.528
	Interaction	F_(1,52)_ = 1.30	0.259
Ripple frequency	Genotype	F_(1,20)_ = 0.43	0.518
	Condition	F_(1,20)_ = 1.01	0.328
	Interaction	F_(1,20)_ = 0.67	0.423
Ripple power	Genotype	F_(1,19)_ = 4.91	0.039
	Condition	F_(1,19)_ = 0.38	0.545
	Interaction	F_(1,19)_ = 0.01	0.910
MUA-Base	Genotype	F_(1,47)_ = 0.32	0.573
	Condition	F_(1,47)_ = 0.04	0.847
	Interaction	F_(1,47)_ = 0.23	0.637
MUA-SWR	Genotype	F_(1,49)_ = 0.92	0.343
	Condition	F_(1,49)_ = 0.05	0.824
	Interaction	F_(1,49)_ = 0.001	0.979

LMMs were run with genotype (WT vs. WV) and condition (control, tertiapin-Q) as factors. Values represent F statistics with the corresponding p-values.

**Figure 4.**
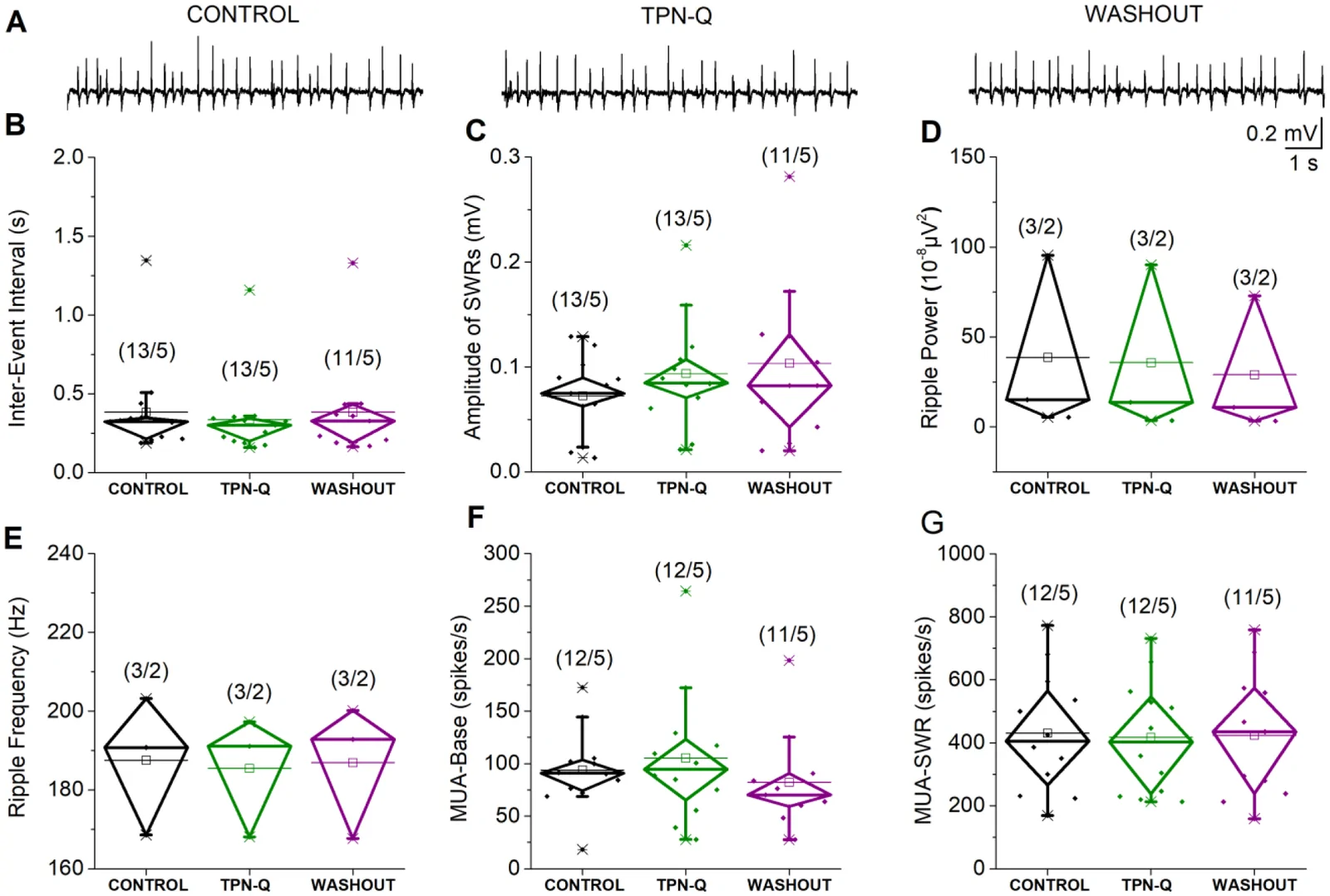
Effects of acute GIRK channel blockade (tertiapin-Q, TPN-Q) on SWR and MUA properties in WT slices. A: Representative traces of spontaneous activity recorded under control conditions, during application of TPN-Q, and after washout. B: IEI. TPN-Q did not significantly alter SWR occurrence compared with control conditions. C: SWR amplitude. No significant effect of TPN-Q was observed. D: Ripple power. No significant effect of TPN-Q was detected. E: Ripple peak frequency. No significant drug effect was observed. F: MUA-Base. No significant drug effect was detected. G: MUA-SWR. TPN-Q did not significantly affect MUA-SWR. Data are presented as diamond plots showing the median, interquartile range (25^th^–75^th^ percentiles), mean, and 5^th^–95^th^ percentiles; individual data points represent slices. Numbers in parentheses indicate the number of slices/animals per condition. Statistical comparisons were performed between control and TPN-Q conditions using LMMs (see Methods). Washout data are shown for descriptive purposes only and were not included in the statistical analysis. Statistical significance was set at p < 0.05.

### 3.7. Effects of GIRK channel blockade on SWR and MUA properties

To assess the contribution of GIRK channel activity to the hippocampal network's dynamics, we examined the effects of the selective GIRK channel blocker tertiapin-Q on SWR properties in WT (Figure 4) and WV (Figure 5) hippocampal slices. Statistical analysis was performed using LMMs with genotype (WT vs WV) and experimental condition (control, tertiapin-Q) as fixed factors. LMM analysis of IEI revealed no significant effects of genotype (F_(1,52)_ = 0.14, p = 0.710), drug condition (control vs tertiapin-Q; F_(1,52)_ = 1.12, p = 0.295), or their interaction (F_(1,52)_ = 0.17, p = 0.681). These results indicate that acute GIRK channel blockade does not significantly alter SWR occurrence under the present experimental conditions. Similarly, analysis of SWR amplitude revealed no significant effects of genotype (F_(1,52)_ = 0.07, p = 0.788), drug condition (F_(1,52)_ = 0.40, p = 0.528), or their interaction (F_(1,52)_ = 1.30, p = 0.259). These findings indicate that acute GIRK channel blockade does not significantly affect the amplitude of SWRs. Analysis of ripple power (log-transformed) revealed a significant main effect of genotype (F_(1,19)_ = 4.91, p = 0.039), with lower ripple power observed in WV slices compared with WT slices. These results are summarized in Table 2. Because this analysis was performed on the subset of slices included in the pharmacological experiments, this finding should be interpreted within the context of that dataset and not as a contradiction of the primary genotype analysis presented in Table 1. There was no significant effect of drug condition (F_(1,19)_ = 0.38, p = 0.545), and no interaction between genotype and condition (F_(1,19)_ = 0.01, p = 0.910) was found, indicating that tertiapin-Q did not significantly influence ripple power in either genotype. Ripple peak frequency was not significantly affected by genotype (F_(1,20)_ = 0.43, p = 0.518), drug condition (F_(1,20)_ = 1.01, p = 0.328), or their interaction (F_(1,20)_ = 0.67, p = 0.423), indicating that neither chronic GIRK disruption nor acute pharmacological blockade alters the frequency of ripple oscillations.

MUA-Base (log-transformed) was not significantly affected by genotype (F_(1,47)_ = 0.32, p = 0.573), drug condition (F_(1,47)_ = 0.04, p = 0.847), or their interaction (F_(1,47)_ = 0.23, p = 0.637). Moreover, MUA-SWR (log-transformed) was not significantly affected by genotype (F_(1,49)_ = 0.92, p = 0.343), drug condition (F_(1,49)_ = 0.05, p = 0.824), or their interaction (F_(1,49)_ = 0.001, p = 0.979), indicating that neither chronic GIRK disruption nor acute pharmacological blockade alters the baseline firing rates or neuronal firing during SWRs. The results of the LMM analyses for the pharmacological experiments are summarized in Table 2.

Taken together, these results indicate that acute GIRK channel blockade does not significantly modulate SWR dynamics in either genotype, nor does it differentially affect WT and WV hippocampal networks.

**Figure 5.**
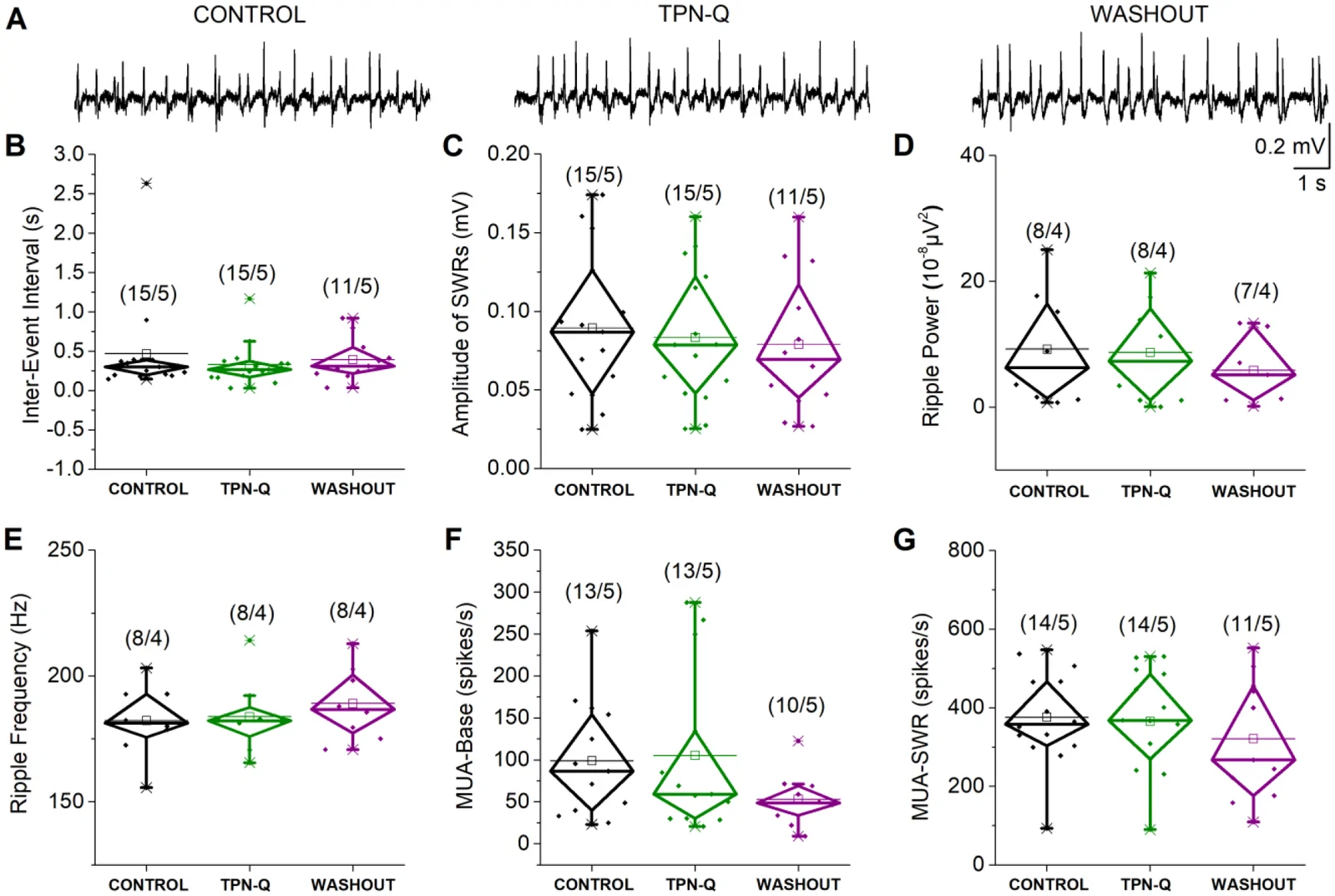
Effects of acute GIRK channel blockade with tertiapin-Q (TPN-Q) on SWR and MUA properties in WV mice. A: Representative field potential recordings under control conditions, during TNP-Q application, and after washout. B: IEI. TPN-Q did not significantly alter SWR occurrence compared with control conditions. C: SWR amplitude. No significant effect of TPN-Q was observed. D: Ripple power. No significant effect of TPN-Q was detected. E: Ripple peak frequency. No significant effect of TPN-Q was observed. F: MUA-Base. No significant effect of TPN-Q was detected. G: MUA-SWR. No significant effect of TPN-Q was observed. Data are presented as diamond plots showing the median, interquartile range (25^th^–75^th^ percentiles), mean, and 5^th^–95^th^ percentiles; individual data points represent slices. Numbers in parentheses indicate the number of slices/animals per condition. Statistical comparisons were performed between control and TPN-Q conditions using LMMs (see Methods). Washout data are shown for descriptive purposes only and were not included in the statistical analysis. Statistical significance was set at p < 0.05.

## 4. Discussion

In the present study, we examined how chronic disruption of GIRK2 signaling in the WV mouse affects hippocampal SWR dynamics along the dorsoventral axis, and whether these effects can be recapitulated by acute pharmacological blockade of GIRK channels. Our results reveal selective alterations of the hippocampal network's activity in the WV model. Specifically, we found that SWR occurrence was primarily determined by dorsoventral location and was not significantly affected by genotype, while SWR amplitude was also region-dependent and preserved across genotypes. Ripple oscillation properties did not show significant genotype-dependent differences. In contrast, MUA-Base was significantly increased in WV mice, whereas MUA-SWR showed a tendency toward reduction. Importantly, acute GIRK blockade with tertiapin-Q did not significantly alter SWR dynamics under the present experimental conditions, nor did it differentially affect WT and WV SWR/MUA. Together, these findings indicate that chronic disruption of GIRK signaling produces selective changes in networks' excitability and neuronal recruitment that are not reproduced by acute pharmacological blockade.

The robust dorsoventral differences in SWR and MUA are in line with previous work demonstrating regional specialization of hippocampal network dynamics [7],[15],[16],[39]. This gradient likely reflects systematic differences in intrinsic excitability, inhibitory circuit organization, and neuromodulatory tone along the dorsoventral axis [11],[14]. In this context, the absence of a significant genotype effect on SWR occurrence indicates that the core mechanisms governing SWR timing are largely preserved in the WV model and remain dominated by the regional network's properties.

Our data reveal a dissociation across different dimensions of the hippocampal network's activity. While SWR incidence and amplitude were not significantly affected by genotype, baseline neuronal firing was significantly increased in WV mice, indicating elevated intrinsic excitability. In contrast, MUA-SWR showed a tendency toward reduction, suggesting impaired recruitment or synchronization of neuronal ensembles during network events. Ripple oscillation properties were largely preserved and did not show consistent genotype-dependent differences across analyses. Together, these findings indicate that chronic disruption of GIRK signaling does not fundamentally alter the generation of SWRs, but instead selectively affects the background excitability of the network and the coordination of neuronal firing during these events. This interpretation is consistent with current models of SWR generation, which emphasize the importance of precisely timed interactions between pyramidal neurons and interneurons in producing ripple oscillations and structured firing patterns [1],[40]–[43].

The cellular distribution of GIRK channels in the hippocampus also provides a framework for interpreting the present findings. GIRK channels exert inhibitory control over neuronal excitability and synaptic integration [24]. In the hippocampus, GIRK2-containing channels are abundantly expressed in CA1 and CA3 pyramidal neurons, where they regulate resting membrane conductance, dendritic integration, and inhibitory neuromodulatory signaling [22],[24],[28]. GIRK subunits are also expressed in several classes of hippocampal interneurons, although their abundance and subunit composition differ among interneuron populations. Consequently, chronic disruption of GIRK2 function is expected to influence both principal neurons and inhibitory interneurons, potentially modifying the balance between excitation and inhibition through multiple cellular mechanisms rather than through alterations confined to a single neuronal population.

Previous work has shown that pharmacological blockade of GIRK channels with tertiapin-Q can increase SWR occurrence under certain conditions, particularly in the dorsal hippocampus [16], suggesting that GIRK-mediated conductance normally restrains networks' excitability. However, in the present study, acute GIRK blockade did not significantly alter SWR parameters in either genotype, nor did it interact with genotype effects. This indicates that the influence of GIRK channels on spontaneous SWR activity is not consistently expressed across experimental conditions. Such discrepancies may reflect species differences (rat vs mouse), differences in the baseline network state, or variability in the slice conditions. Importantly, the lack of effect of tertiapin-Q in WV slices is compatible with the reduced functional availability of GIRK channels in this model, suggesting a form of functional occlusion.

A potential limitation of the present study concerns both the age of the animals and the acute pharmacological protocol used. All experiments were performed in young adult mice (1.5–2 months of age), a developmental stage at which the WV phenotype is well established but at which age-dependent changes in hippocampal physiology may still occur. In addition, the conclusions regarding GIRK channel blockade are restricted to the acute application protocol used here. Although no significant effects of tertiapin-Q were detected under these experimental conditions, different treatment paradigms or application durations were not examined and may produce different outcomes. Therefore, the present findings should be interpreted within the context of the experimental conditions used in this study. An additional consideration is that tertiapin-Q is not selective for GIRK2-containing channels. Rather, its inhibitory potency depends on the molecular composition of GIRK channel complexes, showing greater activity toward GIRK channels containing GIRK1 subunits. Consequently, the pharmacological experiments performed here should not be regarded as a direct functional equivalent of the WV mutation, which specifically disrupts GIRK2 function throughout development. Instead, the comparison between acute tertiapin-Q application and the WV phenotype should be interpreted as contrasting acute pharmacological inhibition of tertiapin-Q-sensitive GIRK channels with the long-term consequences of chronic GIRK2 dysfunction and the associated network adaptations.

An additional limitation of the present study is that recordings from the dorsal and ventral hippocampus were pooled in the pharmacological experiments. Because the number of recordings available from each region separately was limited, the study was not designed or sufficiently powered to evaluate any region-specific effects of tertiapin-Q. Consequently, subtle dorsoventral differences in the pharmacological response cannot be excluded. Future studies with larger numbers of recordings from each hippocampal segment will be required to determine whether acute GIRK channel blockade differentially modulates dorsal and ventral hippocampal networks.

The present observations indicate that the WV phenotype cannot be fully explained by a simple loss of GIRK-mediated conductance but instead reflects selective reorganization of the hippocampal circuits. The WV mutation produces widespread developmental and neurophysiological alterations, including disrupted neuronal migration and long-term changes in synaptic organization [31]–[33]. Accumulating evidence indicates that genetic or developmental perturbations can trigger homeostatic and compensatory plasticity mechanisms acting across multiple levels, from synaptic scaling to circuit reorganization, to stabilize overall activity [34],[35],[44]–[48]. In this context, the observed phenotype likely reflects not only the direct loss of GIRK-mediated conductance but also emergent properties of reorganized hippocampal networks. Although the WV mutation affects multiple brain regions, including structures interconnected with the hippocampus, the present recordings were obtained from isolated hippocampal slices, in which ongoing influences from extra-hippocampal circuits were eliminated. Consequently, the observed alterations in SWR dynamics and neuronal activity reflect intrinsic properties of the hippocampal network. Nevertheless, we cannot exclude the possibility that developmental abnormalities occurring elsewhere in the brain contributed indirectly to the long-term maturation and organization of hippocampal circuits before slice preparation. Such developmental influences are inherent to genetic models such as the WV mouse and cannot be dissociated from the chronic effects of GIRK2 dysfunction in the present experimental design.

More generally, the present findings link ion channel dysfunction to emergent network dynamics with potential functional implications. GIRK channels regulate neuronal excitability and synaptic integration [24], and their disruption leads to altered circuit behavior [27]. Because SWRs depend on a finely tuned excitation–inhibition balance and coordinated pyramidal–interneuron interactions [49],[50], even subtle changes in intrinsic conductances can influence the coordination of neuronal ensembles without necessarily altering the occurrence of network events [1],[40],[51]. Importantly, SWRs play a causal role in memory consolidation and cognitive processing [1],[52]–[55]. In this context, the increase in baseline excitability observed in the WV model, together with a tendency toward reduced neuronal recruitment during SWRs, may reflect subtle alterations in the temporal organization of network activity that could influence memory-related processes.

## 5. Conclusions

In conclusion, chronic GIRK2 disruption in the WV model preserves the fundamental structure of hippocampal SWRs while selectively altering baseline excitability and the coordination of neuronal activity during network events. The absence of comparable effects following acute GIRK blockade indicates that these changes arise from long-term circuit adaptations rather than direct channel function alone. These findings show how ion channel dysfunction can reshape network dynamics without disrupting the core oscillatory patterns, and they emphasize the importance of distinguishing between direct channel effects and compensatory network reorganization when linking molecular perturbations to emergent brain activity, with potential implications for understanding circuit-level alterations in neurological disorders.

## Use of AI tools declaration

The authors declare they have not used artificial intelligence (AI) tools in the creation of this article.


