# Supplementary material for: Chronic GIRK2 disruption in the weaver mouse selectively alters hippocampal excitability without affecting sharp wave-ripple dynamics
Source: AIMS Neurosci. 2026 Sep 9;13(3):530–49. doi: 10.3934/Neuroscience.2026024 (PMC13658092; doi:10.3934/Neuroscience.2026024)
Supplement: Supplementary file 1 [file neurosci-13-03-024-s001.pdf]

AIMS Neuroscience, 13(3): 530–549.
DOI: 10.3934/Neuroscience.2026024
Received: 06 May 2026
Revised: 04 July 2026
Accepted: 24 July 2026
Published: 09 September 2026

https://www.aimspress.com/journal/neuroscience

Research article

Chronic GIRK2 disruption in the weaver mouse selectively alters hippocampal excitability without affecting sharp wave-ripple dynamics

Giota Tsotsokou[1], Vasiliki Sarantopoulou[1] and Costas Papatheodoropoulos[1,2,]*

[1] Department of Medicine, Laboratory of Physiology, University of Patras, Greece
[2] School of Health Sciences, Laboratory of Experimental Animals, University of Patras, Greece

* **Correspondence:** Email: cepapath@upatras.gr; Tel: +302610969117.

Supplementary

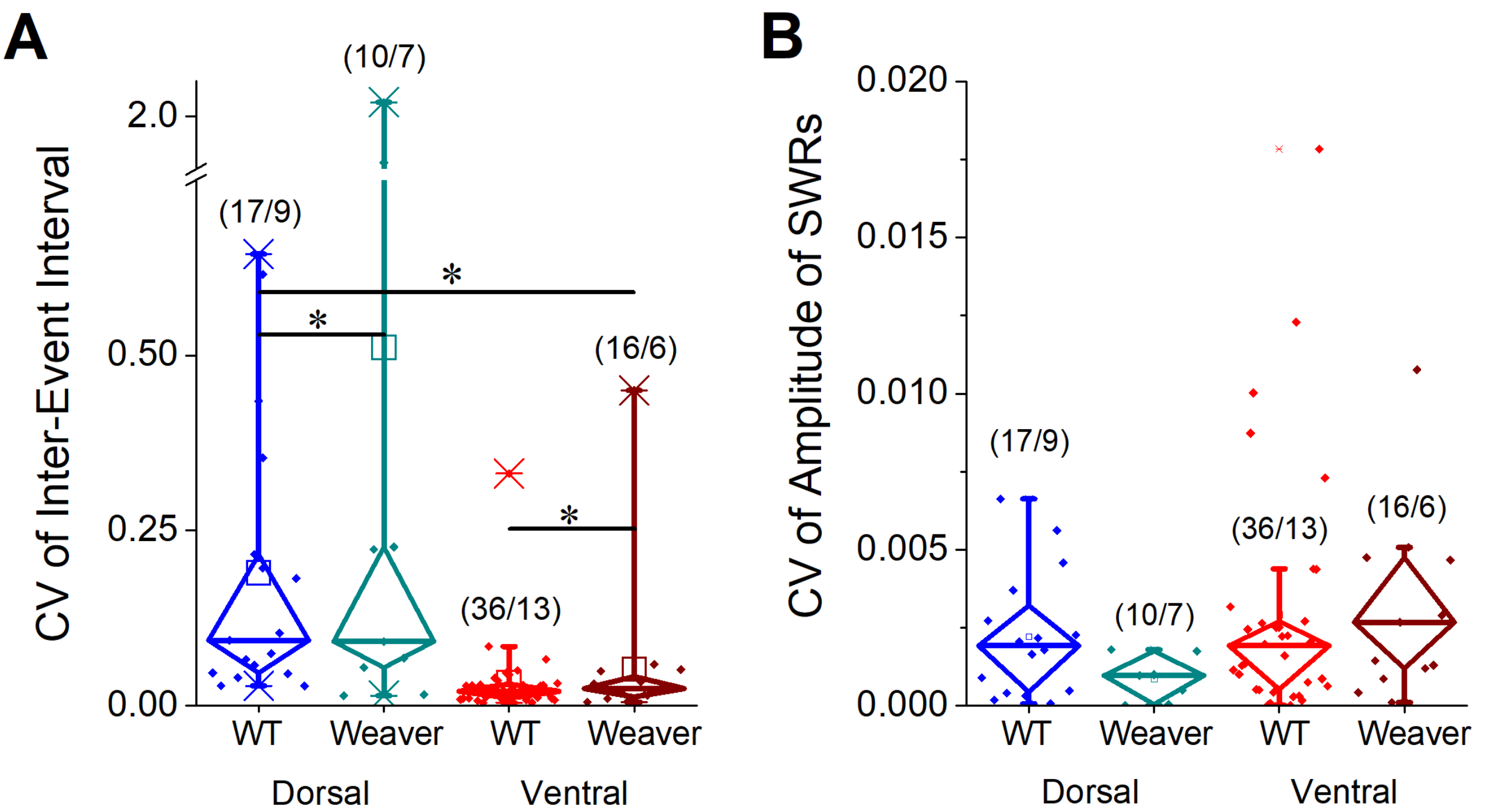

Supplementary Figure S1. Variability of spontaneous SWR occurrence and amplitude. A. Coefficient of variation (CV) of inter-event intervals measured for each hippocampal slice from dorsal and ventral hippocampus of WT and weaver mice. The CV of inter-event intervals differed significantly between dorsal and ventral hippocampus, whereas no significant genotype or interaction between genotype and region was detected. B. Coefficient of variation of SWR amplitude. No significant effects of genotype, hippocampal region, or their interaction were observed. Data are presented as diamond plots showing the median, interquartile range, mean, 5th–95th percentiles, and outliers. Numbers above the plots indicate slices/animals.

AIMS Press

© 2026 the Author(s), licensee AIMS Press. This is an open access article distributed under the terms of the Creative Commons Attribution License (https://creativecommons.org/licenses/by/4.0)
